# Comparison of the Immune Response in Vaccinated People Positive and Negative to SARS-CoV-2 Employing FTIR Spectroscopy

**DOI:** 10.3390/cells11233884

**Published:** 2022-12-01

**Authors:** Gustavo Jesus Vazquez-Zapien, Adriana Martinez-Cuazitl, Miguel Sanchez-Brito, Raul Jacobo Delgado-Macuil, Consuelo Atriano-Colorado, Francisco Garibay-Gonzalez, Virginia Sanchez-Monroy, Alberto Lopez-Reyes, Monica Maribel Mata-Miranda

**Affiliations:** 1 Escuela Militar de Medicina, Centro Militar de Ciencias de la Salud, Secretaría de la Defensa Nacional, Mexico City 11200, Mexico; 2 Escuela Nacional de Medicina y Homeopatía, Instituto Politécnico Nacional, Mexico City 07320, Mexico; 3 Escuela Superior de Cómputo, Instituto Politécnico Nacional, Mexico City 07738, Mexico; 4 Centro de Investigación en Biotecnología Aplicada, Instituto Politécnico Nacional, Santa Inés Tecuexcomac 90700, Mexico; 5 Escuela Superior de Medicina, Instituto Politécnico Nacional, Mexico City 11340, Mexico; 6 Instituto Nacional de Rehabilitación Luis Guillermo Ibarra Ibarra, Secretaría de Salud, Mexico City 14389, Mexico

**Keywords:** COVID-19, SARS-CoV-2, vaccine, FTIR spectroscopy, immunological response

## Abstract

Various immunopathological events characterize the systemic acute respiratory syndrome coronavirus 2 (SARS-CoV-2) infection. Moreover, it has been reported that coronavirus disease 2019 (COVID-19) vaccination and infection by SARS-CoV-2 induce humoral immunity mediated by B-cell-derived antibodies and cellular immunity mediated by T cells and memory B cells. Immunoglobulins, cytokines, and chemokines play an important role in shaping immunity in response to infection and vaccination. Furthermore, different vaccines have been developed to prevent COVID-19. Therefore, this research aimed to analyze and compare Fourier-transform infrared (FTIR) spectra of vaccinated people with a positive (V-COVID-19 group) or negative (V-Healthy group) real-time quantitative reverse transcription-polymerase chain reaction (RT-qPCR) test, evaluating the immunoglobulin and cytokine content as an immunological response through FTIR spectroscopy. Most individuals that integrated the V-Healthy group (88.1%) were asymptomatic; on the contrary, only 28% of the V-COVID-19 group was asymptomatic. Likewise, 68% of the V-COVID-19 group had at least one coexisting illness. Regarding the immunological response analyzed through FTIR spectroscopy, the V-COVID-19 group showed a greater immunoglobulins G, A, and M (IgG, IgA, and IgM) content, as well as the analyzed cytokines interferon-gamma (IFN-γ), tumor necrosis factor-alpha (TNF-ɑ), and interleukins 1β, 6, and 10 (IL-1β, IL-6, and IL-10). Therefore, we can state that it was possible to detect biochemical changes through FTIR spectroscopy associated with COVID-19 immune response in vaccinated people.

## 1. Introduction

Systemic acute respiratory syndrome coronavirus 2 (SARS-CoV-2) was first described in November 2002 in Guangdong, China. At that time, it spread rapidly worldwide to 29 countries. Then, at the end of 2019, China declared an epidemic of a novel coronavirus, SARS-CoV-2, which provoked the coronavirus disease 2019 (COVID-19). On 11 March 2020, the World Health Organization (WHO) considered COVID-19 a pandemic, taking many people’s lives to this day [1,2].

The high transmissibility, presence of asymptomatic carriers, fast spread of the virus, and the emergence of new variants and mutations became a global problem. As a result, scientists and research institutes made inroads into treatment strategies. Moreover, aiming for the prevention of COVID-19, different vaccines were developed, including viral-like particle vaccines, entire inactivated virus vaccines, viral vector vaccines, live attenuated virus vaccines, subunit vaccines, RNA vaccines, and DNA vaccines [3,4]. Nowadays, vaccination constitutes the most promising path back to ‘normal life’ [5].

It has been reported that COVID-19 vaccination and infection by SARS-CoV-2 induce humoral immunity mediated by B-cell-derived antibodies and cellular immunity mediated by T cells and memory B cells [6]. In addition, SARS-CoV-2 infection is characterized by various immunopathological events, such as cytokine storm, lymphocyte activation and dysfunction, increased level of neutrophils, and depletion and exhaustion of lymphocytes, promoting a significant immune response, including immune activation and antiviral immune response. However, the exact nature of effective immunity still needs to be defined [7,8].

It is known that an immune response begins when macrophages ingest antigens and digest them into antigen fragments. The major histocompatibility complex carries some of these fragments to the cell’s surface, where they are displayed and recognized by T cells, stimulating B cells to secrete antibodies to the fragments and prompt other immune defenses [9]. The response to viral infection can be categorized into innate (non-specific defense mechanisms) and adaptive (specific defense mechanisms) immunity [10]. Even though the innate and adaptive immune systems have been considered as contrasting arms, they usually act together [11].

The innate immune response is the first line of host defense against viral infection, including SARS-CoV-2, and becomes prominent after several days. This tries to limit viral entry, translation, replication, and assembly, through physical barriers, soluble proteins, and small bioactive molecules that are either constitutively present in biological fluids (such as the complement proteins, defensins, and ficolins) or that are released from cells as they are activated (including cytokines, chemokines, lipid mediators of inflammation, among others) [11]. Many viruses activate the innate immune system through TLR and most TLRs use MyD88 to trigger inflammatory cytokine production, including tumor necrosis factor (TNF), IL-6, and IL-1 [12]. In summary, the innate immune response serves three main purposes: (1) restriction of viral replication, (2) creation of an antiviral state in the local tissue environment, and (3) priming the adaptive immune response [13], highlighting that IL-6, TNF-α, MIP-1α, and IP-10 are produced by innate immune cells, such as macrophages and natural killer cells, in response to pathogen recognition. However, it is essential to mention that TNF-α and IL-6 are critical cytokines in developing immune responses against viral vaccines [14]. Particularly, IL-6 is considered a crucial cytokine for developing an antigen-specific humoral response during some infections, and IL-10 is an important anti-inflammatory cytokine that downregulates the production of pro-inflammatory cytokines and generally protects from systemic inflammation [15].

In the same way, the adaptive response is essential to control viral infections. It is based primarily on the antigen-specific receptors expressed on the surfaces of T- and B-lymphocytes [11]. There are two classes of adaptive responses: antibody responses, where B cells are activated to secrete antibodies, called immunoglobulins, and cell-mediated immune responses, where activated T cells react directly against a foreign antigen that is presented to them on the surface of a host cell [16]. Therefore, there are three fundamental components in the adaptive immune system: B cells, CD4+ T cells, and CD8+ T cells. However, contrary to the innate immune response, the adaptive response takes time to generate sufficient cells to control viral infection, ∼6–10 days after priming [13].

During SARS-CoV-2 infection, the production of specific antibodies against the virus is consistent in most patients; IgM can be found as early as three days after infection and provides the first line of antibodies in immunity defense. After that, IgG responses are initiated and play a key role in long-term immune memory [17].

On the other hand, it is known that SARS-CoV-2-specific T-cell responses are critical for COVID-19 protection [18]. Furthermore, it has been reported that CD4+ T-cell responses to SARS-CoV-2 are more prominent than CD8+ T-cell responses and are associated with controlling primary SARS-CoV-2 infection [13], making the amount of T-cell response a powerful tool to measure potential immunity from COVID-19 [7,18], highlighting that CD4+ response is characterized by the production of interferon-gamma (IFN-γ), tumor necrosis factor-alpha (TNF-ɑ), and interleukin-2 (IL-2) [14].

As previously mentioned, immunoglobulins, cytokines, and chemokines play an important role in shaping immunity in response to infection and vaccination, and some authors have declared that patients with severe COVID-19 have substantially increased IL-1β, IL-2, IL-6, IL-7, IL-8, IL-10, IL-17, IFN-γ, G-CSF, and TNF-α, showing a predictive effect [19,20].

On the other hand, even though the pandemic’s spread seems to be controlled, an affordable COVID-19 rapid and reliable diagnostic test for screening is needed to prevent its resurgence in areas preparing for a return to economic activities, and current diagnostic tests are not helpful for this purpose. Although real-time quantitative reverse transcription-polymerase chain reaction (qRT-PCR) is the gold standard for molecular diagnosis of COVID-19, it requires expensive qPCR instruments and experienced laboratory technologists. Antigen rapid diagnostic tests for COVID-19 proved to be 76.3% sensitive and 99.1% specific, with higher sensitivity in symptomatic compared to asymptomatic persons, especially at the first week of symptom onset [21]. On the other hand, an antibody test has also been used as a rapid diagnostic test. However, positive antibodies are only detected after 7–14 days and cannot differentiate between acute and past infection. Therefore, they do not help identify actively infected COVID-19 patients [22].

In a previous work [23], we proposed the use of Fourier-transformed infrared spectroscopy (FTIR) and the employment of artificial intelligence (AI) methods to discriminate healthy from COVID-19-infected people. Nevertheless, most of the population had not received vaccines at that time. Therefore, considering the vaccination campaign worldwide and the unknown contact with the virus, it seems interesting to look for the immune response after vaccination through FTIR spectroscopy. Therefore, this research aimed to analyze and compare FTIR spectra of vaccinated patients with a positive qPCR test vs. another group formed by vaccinated people with a negative qPCR test, evaluating the immune response through FTIR spectroscopy, highlighting that, in the future, with the results obtained herein, more AI methods should be tested to discriminate these populations.

## 2. Materials and Methods

### 2.1. Sample Collection

The population used for this research was divided into two groups. The V-COVID-19 group was composed of vaccinated people who had reported COVID-19 symptoms such as dyspnea, cough, headache, runny nose, and fever, among others, in the last three days with a positive qPCR test for COVID-19 in this period. The V-Healthy group was composed of vaccinated people without COVID-19 symptoms. In addition, at the time of saliva sampling, oropharyngeal swabs were taken to demonstrate the absence of COVID-19 using a qPCR test.

Saliva samples were collected from September 2021 to March 2022 in the “Hospital Central Militar” of the National Defense Ministry—Mexico. The inclusion criteria were patients who agreed to participate in this research, aged over 18 years, vaccinated no longer than one year ago, had a fasting period of 8 h, and had a positive or negative qPCR test within the previous five days of the sample collection. The considered exclusion criteria were patients with orthodontic or other dental treatment and patients who had rinsed or brushed the oral cavity with mouthwash before sampling.

People in both groups were asked to donate 1 mL of saliva collected in sterile 1.5 mL microcentrifuge tubes. Samples were maintained in the cold (4 °C) until their analysis was carried out in the following three hours. Personal protective equipment was used by the personnel in charge of sampling and analyzing the samples that integrated the V-COVID-19 group.

The appropriate ethics committee approved and examined all experiments; the ethical standards in the 1964 Declaration of Helsinki were followed. In the same way, the Institutional Human Research Ethical Committee approved the protocol and the written informed consent forms.

### 2.2. Medical Data Collection

When the samples were collected, symptoms (cough, dyspnoea, headache, and fever, among others) and comorbidities, such as diabetes, obesity, hypertension, and asthma were examined; data were expressed in frequency and percentage. Finally, in the V-COVID-19 group, laboratory blood tests (hematic biometry, blood chemistry test, hepatic function test, blood gas test, and others) were evaluated, obtaining the average and the interquartile range (IQR) of each studied parameter.

### 2.3. Saliva Sample Pre-Processing and Spectral Analysis

The FTIR spectra analysis was conducted in a spectral range between 4000 and 400 cm^−1^ using an FTIR spectrometer (6600, Jasco, Tokyo, Japan); the instrument has a fixed spectral resolution of 4 cm^−1^. For the sample analysis, three μL from the saliva sample was placed onto the attenuated total reflectance (ATR) crystal; subsequently, the sample was allowed to dry at room temperature for about 15 min to eliminate excess water. Once the band attributed to water was not detected, the spectra were captured 120 times, and the average of data acquisition was developed to obtain one spectrum. Each sample was analyzed in triplicate.

The spectra were normalized through standard normal variate (SNV) and the analysis was performed in the biological fingerprint region (1800–800 cm^−1^). After that, the mean of each population was obtained to identify relevant differences (absorbance differences and displacements). The graphs were obtained using Origin software (version 8.5, OriginLab Corporation, Northampton, MA, USA).

### 2.4. Immune Response Analysis through FTIR Spectroscopy

The study of the immune response analysis through FTIR considered immunoglobulins such as IgG, IgA, and IgM, as well as some cytokines, including IFN-γ, TNF-α, IL-1 β, IL-6, and IL-10.

For the IgG, IgA, and IgM analysis, we employed bands that other authors had previously reported, such as Benezzeddine–Boussaidi, who attributed the bands at 1560–1464 cm^−1^ and 1285–1237 cm^−1^ to IgG and IgA, respectively, and at 1420–1289 cm^−1^ and 1160–1028 cm^−1^ to IgM [23,24].

On the other hand, for the cytokine analysis, we analyzed different cytokines in their pure form at different concentrations to establish the specific spectra of each one of them, as follows: IFN-γ (300-02, Preprotech, Cranbury, NJ, USA), TNF-α (300-01A, Preprotech, Cranbury, NJ, USA), IL-1 β (575109, Biolegend, San Diego, CA, USA), IL-6 (900-M16, Preprotech, Cranbury, NJ, USA), and IL-10 (575809, Biolegend, San Diego, CA, USA). Then, the obtained spectra were normalized through SNV and averaged. After that, the significant bands associated with the studied cytokine were identified, as well as the bands that increased its absorbance with higher concentrations. Then, the most representative bands for each cytokine were attributed to that cytokine.

Finally, employing the bands related to each immunoglobulin and cytokine, the integrated areas of the V-COVID-19 and V-Healthy groups were developed and a U-Mann–Whitney test was used to determine significant differences between groups using Graph Pad 8.0.

## 3. Results

In this work, we analyzed the FTIR spectra of vaccinated people grouped into the V-COVID-19 and V-Healthy groups to identify and compare biochemical changes between the populations by studying the immune response through FTIR spectroscopy.

### 3.1. Integration of the Population

The V-COVID-19 group included 50 patients and the V-Healthy group had 135 persons, distributed by gender, as shown in Table 1. The average age of the V-COVID-19 group was 52.2 ± 17 years and for the V-Healthy group, 48 ± 15 years.

As previously mentioned, the population included vaccinated people who received at least one dose of BioNTech, Cansino, CoronaVac, Covaxin, Johnson & Johnson, AstraZeneca, or Sputnik vaccine, as seen in Table 2, where it can be observed that the most frequently used vaccines were AstraZeneca, Cansino, and BioNTech in both groups.

**Figure 1:**
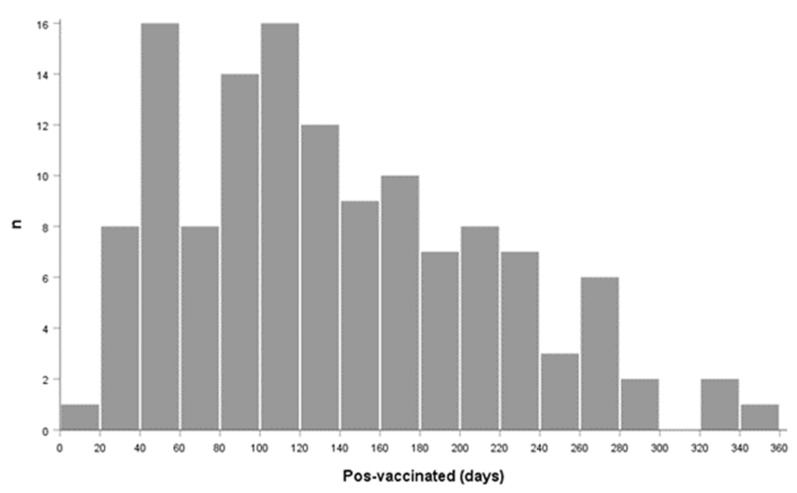
Frequency of the days elapsed between the last vaccine dose and sampling.

The days elapsed between the last vaccine dose were from 17 up to 357 days, as seen in Table 3 and depicted in Figure 1, where it is observed that the majority of the population received the vaccine between 40 and 60 days and 100 and 120 days before sampling.

### 3.2. Analysis of Medical Data

Table 4 outlines the symptoms shown in both groups. In the same way, Table 5 outlines the comorbidities (diabetes, hypertension, obesity, overweight, asthma, chronic obstructive pulmonary disease (COPD), chronic kidney disease (CKD), and other comorbidities, including cervical and pancreatic cancer, as well as psoriasis, dyslipidemia, and Barrett’s esophagus). The common symptoms in the overall V-COVID-19 population were dyspnea, cough, headache, and runny nose, showing statistical significance. In addition, most individuals included in the V-Healthy group (88.1%) were asymptomatic; in contrast, only 28% of the V-COVID-19 group was asymptomatic.

In the same way, 68% of the individuals included in the V-COVID-19 group had at least one coexisting illness, such as hypertension (42%), diabetes (32%), and obesity (8%). In contrast, in the V-Healthy group, only 23.7% and 11.9% of the population referred to hypertension and diabetes, respectively, displaying statistical significance. Likewise, it is essential to mention that only one patient with COVID-19 died, a male at 72 years old, with diabetes and hypertension, and he was vaccinated with two doses of AstraZeneca.

The laboratory blood tests of the V-COVID-19 group are summarized in Table 6, where the median values of leucocytes, neutrophils, lymphocytes, hemoglobin, platelets, and glucose, among other metabolites, can be observed, highlighting that PaO2 was considerably diminished; contrarily, PaCO2 and HCO3- were slightly increased. In the same way, it is essential to mention that the values of C-reactive protein (CRP), fibrinogen, D-dimer, and ferritin were increased.

### 3.3. Saliva FTIR Spectra Analysis and Immune Response

**Figure 2:**
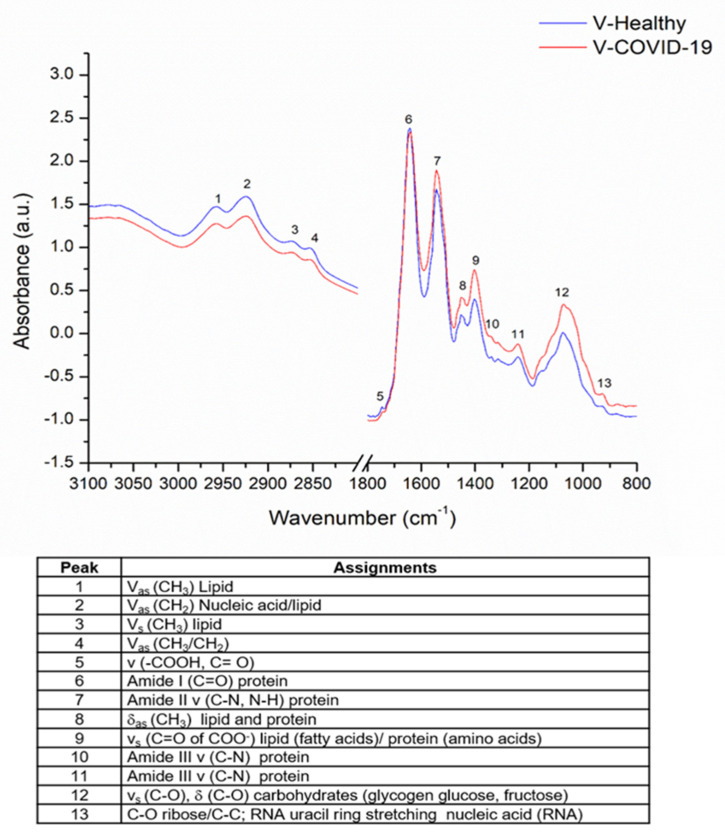
Mean of FTIR spectra of V-COVID-19 (*n* = 50) and V-Healthy (*n* = 135) groups. The spectra of the V-COVID-19 group are represented in red and the spectra of the V-Healthy group are in blue.

The FTIR spectra obtained and averaged are depicted in Figure 2; the spectra of the V-COVID-19 group are represented in red and the spectra of the V-Healthy group are in blue. Different absorption bands related to biological samples, such as lipids, proteins, carbohydrates, and nucleic acids, are evidenced. In Figure 2, each assignment to the detected bands is presented [25,26,27]. Herein, the bands associated with amide II, lipid and protein, lipid/protein, amide III proteins, and carbohydrates showed a greater absorbance in the V-COVID-19 group.

**Figure 3:**
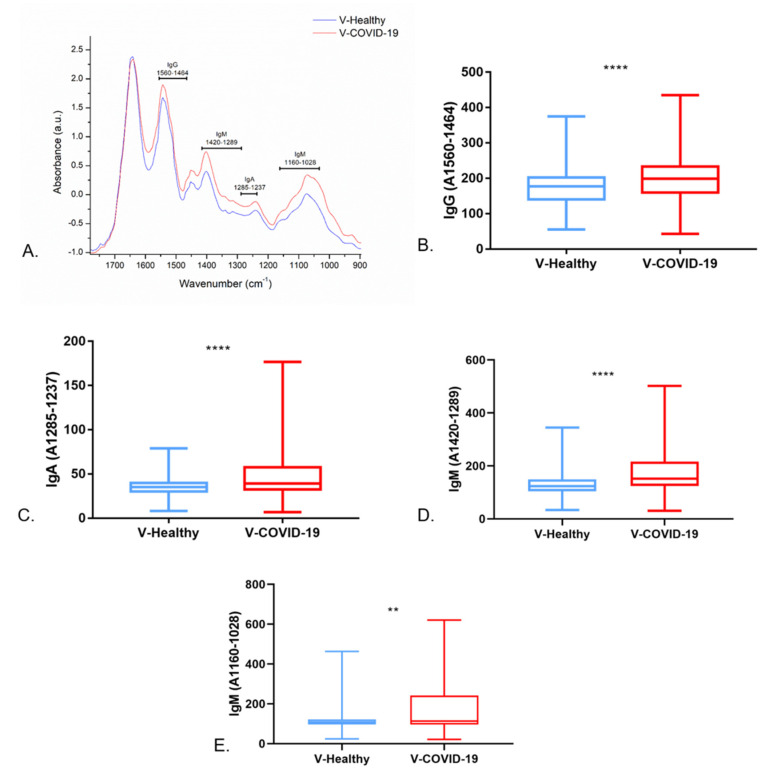
Immunoglobulins content analysis studied through FTIR. Identification of immunoglobulins in the FTIR spectra of saliva samples (**A**), integrated areas of IgG (**B**), integrated areas of IgA (**C**), and integrated areas of IgM (**D**,**E**). ** *p* < 0.05 and **** *p* < 0.0005.

According to previous research, IgG, IgA, and IgM were analyzed in the regions at 1560–1464 cm^−1^ and 1285–1237 cm^−1^ to IgG and IgA, respectively, and at 1420–1289 cm^−1^ and 1160–1028 cm^−1^ to IgM [23,24]. In Figure 3A, it can be observed that the V-COVID-19 group showed a greater absorbance in these regions. Moreover, the integrated areas of IgG, IgA, and IgM evidenced that the V-COVID-19 group showed a greater content of these immunoglobulins (Figure 3B–E), showing statistical significance.

**Figure 4:**
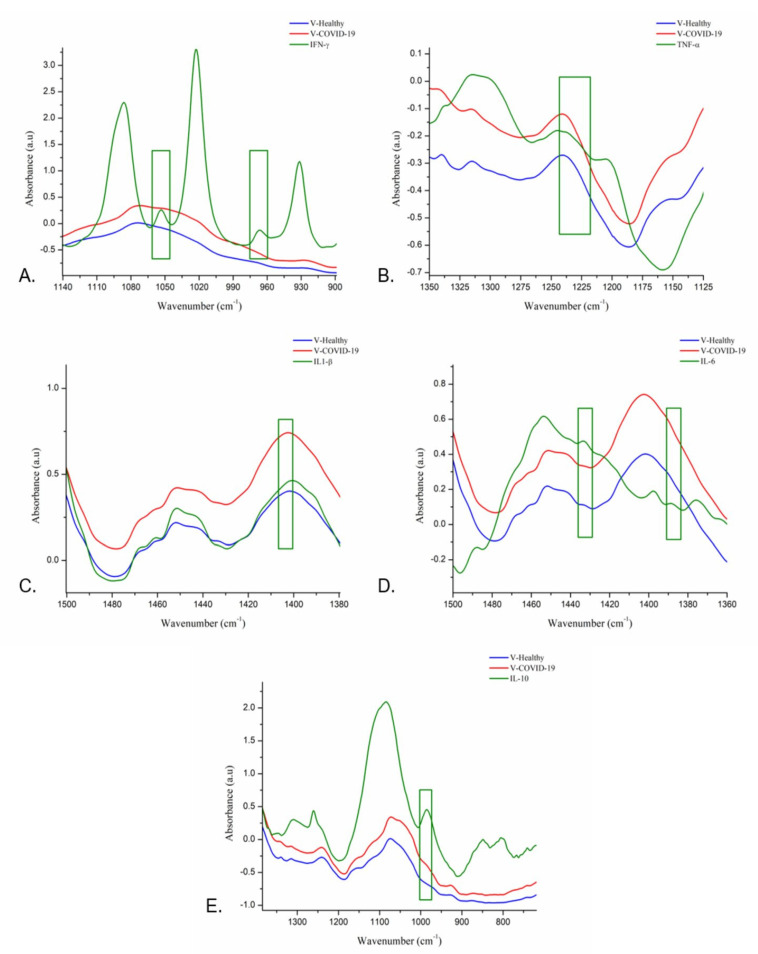
Cytokine analyses through FTIR. The regions at 978–956 cm^−1^ and 1061–1044 cm^−1^ are associated with IFN-γ (**A**), the regions at 1243–1217 cm^−1^ and 1409–1399 cm^−1^ are related to TNF-α (**B**) and IL-1 β (**C**), respectively, and the regions at 1393–1381 cm^−1^ and 1436–1428 cm^−1^ are linked to IL-6 (**D**) and IL-10 (**E**).

The cytokine analysis evidenced that the regions at 1061–1044 cm^−1^ and 978–956 cm^−1^ are associated with IFN-γ; the regions at 1243–1217 cm^−1^ and 1409–1399 cm^−1^ are related to TNF-α and IL-1 β, respectively. In the same way, the regions at 1393–1381 cm^−1^ and 1436–1428 cm^−1^ are linked to IL-6 and IL-10 (Figure 4). Furthermore, in each band related to the cytokines mentioned above, the V-COVID-19 group showed a greater absorbance than the V-Healthy group.

**Figure 5:**
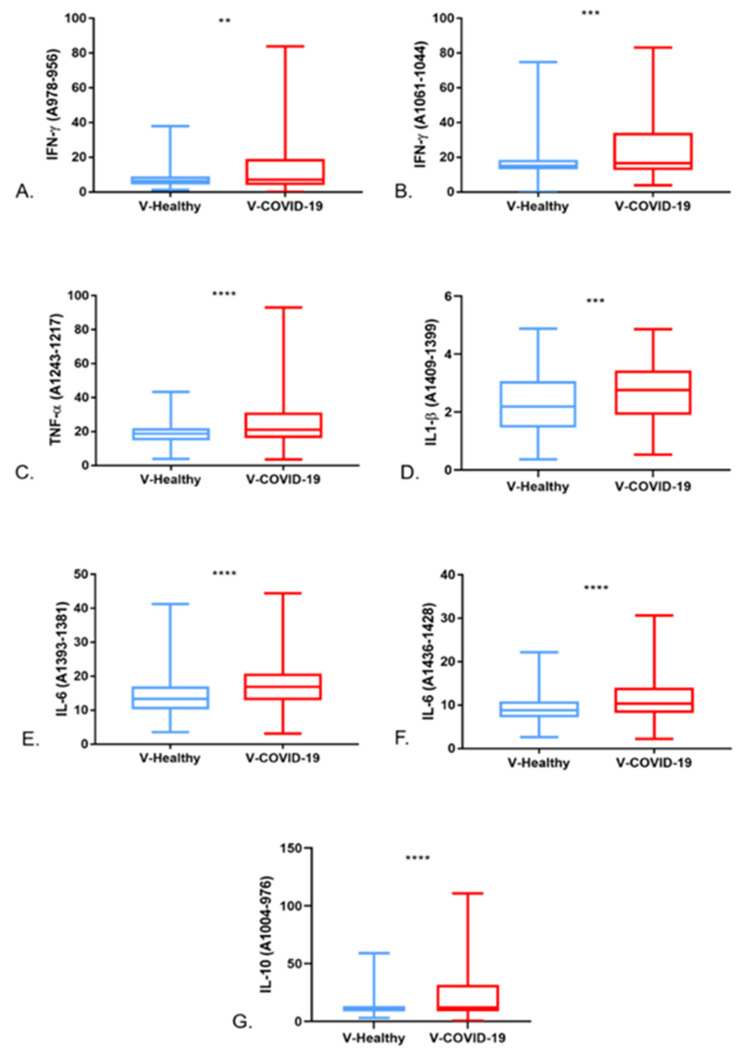
Integrated areas of the regions related to cytokines. IFN-γ (**A**,**B**), TNF-α (**C**), IL-1β (**D**), IL-6 (**E**,**F**), and IL-10 (**G**). ** *p* < 0.05, *** *p* < 0.005 and **** *p* < 0.0005.

The integrated areas of the regions related to IFN-γ, TNF-α, IL-1 β, IL-6, and IL-10 were obtained to elucidate the quantity of those cytokines in the V-COVID-19 and V-Healthy groups. As seen in Figure 5, the V-COVID-19 group showed a more significant amount of all the analyzed cytokines, showing statistical significance. 

## 4. Discussion

In a previous work [23], we reported that using saliva samples analyzed through FTIR and AI techniques could discriminate COVID-19 patients from healthy people; however, with the vaccine advent, biochemical changes related to immunological response were detected in the saliva, bringing about the need to search for these new biochemical changes, as well as the immune response through this vibrational technique.

To achieve the aim of this work, we recruited vaccinated people and integrated two study groups, V-COVID-19 and V-Healthy groups, analyzing their saliva through FTIR spectroscopy and looking for bands associated with immune response.

According to the COVID-19 vaccine tracker on April 2022, there were ten vaccines approved for use in Mexico, including BNT162b2 by Pfizer, Inc./BioNTech, AZD1222 by AstraZeneca/Oxford University, Sputnik V by the Gamaleya Institute, Ad5-nCoV by CanSino Biologics Inc., CoronaVac by Sinovac Research and Development Co., among others. As of 25 November 2021, the AstraZeneca vaccine was the most purchased SARS-CoV-2 vaccine in Mexico, registering almost 80 million purchased doses, followed by Moderna with 39 million, CanSino Biologics (35 million), and Pfizer (34.4 million) [28], which agrees with the obtained results, since the most frequently reported vaccines in this study were AstraZeneca, CanSino Biologics, and Pfizer.

As previously reported in Martinez-Cuazitl et al., 2021 [23], in this research, the most reported symptoms were dyspnea, cough, and headache, and the main comorbidities were hypertension, diabetes, and obesity. In the same way, as expected, the laboratory blood tests evidenced a diminished PaO2 and a slight increase in PaCO2 and HCO3-. Likewise, CRP, fibrinogen, D-dimer, and ferritin were increased. Regarding this, Huang et al. developed a meta-analysis showing that elevated serum CRP, PCT, D-dimer, and serum ferritin levels were associated with higher mortality, severe COVID-19 clinical manifestations, and the need for ICU care. However, the effect estimate was not significantly modified by gender, age, cardiovascular disease, or diabetes [29], which agrees with the results presented in this research, once the population in the V-COVID-19 group was hospitalized.

Regarding the FTIR biochemical analysis, the same bands reported in the previous work [23] were detected and also discussed. However, as previously mentioned, this research’s central aim was looking for the immune response after vaccination through FTIR analysis. Regarding the immunoglobulin analysis, it has been reported that vaccination increases the IgG, IgA, and IgM levels after the first dose. Moreover, previously infected subjects exhibit IgG and IgA detectable levels even after eight months post-infection [30]. 

On the other hand, the major SARS-CoV-2 antigenic target of human IgG and IgA is the spike protein encoded by the mRNA of vaccines [31]. It is known that the BNT162b2 by Pfizer vaccine induces the formation of neutralizing antibodies ten days after the first dose, also inducing IgG elevation for at least 180 days after the first dose, being higher than this expression in previously infected patients; moreover, IgA levels are only detected above the cut-off level from 21 days up to 90 days after the first dose of the vaccine [32,33].

Likewise, Huang et al. reported that IgG antibody titers are stable in saliva samples up to 15 weeks after infection [29]. Moreover, Collier et al. stated that vaccinated infected individuals exhibited markedly higher serum and mucosal antibody responses and cellular immune responses than vaccinated uninfected individuals [34], which was consistent with the results obtained herein. In this research, individuals in both groups were observed 17–357 days after the vaccine, observing that the IgG, IgA, and IgM content was higher in the V-COVID-19 group than in the V-Healthy group, which agrees with that mentioned above.

Regarding cytokine analysis, Pourgholaminejad et al. and Guo et al. stated that cytokine release syndrome is associated with disease severity, characterized by increased TNF-α, IL-1β, IL-6, IL-2, IL-7, IL-8, and IL-10 [19,35]. In this study, we also found an increase in IFN-γ, TNF-α, IL-1 β, IL-6, and IL-10 in the V-COVID-19 group, which included patients that required hospitalization.

It is known that CD4+ T-cell response is heavily responsive toward secretion of Th1 cytokines, specifically IL-2 and IFN-γ [7]. Its activations respond to antigen presentation, detected from day 2–4 after the onset of symptoms, showing the highest levels of IFN-γ between 3 and 6 months, persisting up to 15 months [36]. In the same way, IL-10 directly expands cytotoxic effector CD8+ T-cells in human studies, provoking a hyperactivation of adaptive immunity in COVID-19 patients, which might contribute to exacerbating disease severity. Furthermore, Lu et al. reported that IL-10 concentrations were significantly higher in intensive-care-unit COVID-19 patients compared to non-ICU patients [37]. Therefore, in this research, we observed that IFN- γ and IL-10 showed a higher absorbance and content in the V-COVID-19 group, which agrees with that mentioned above. Furthermore, Lu et al. also declared that IL-10 concentrations strongly correlated with those of IL-6 and other inflammatory markers, such as CRP [37], highlighting that, herein, we also found a high CRP serum concentration in the V-COVID-19 group as well as IL-6, which will be discussed later.

Finally, it is important to mention that SARS-CoV-2 activates the NLRP3 inflammasome, resulting in the processing and secretion of active IL-1β and IL-18 and initiating cytokine release syndrome. In addition, IL-1β induces IL-6 and a Th-17 immune response [38]. Once the nucleocapsid (N) protein of SARS-CoV activates IL-6 expression through the nuclear factor-kappa B (NF-κB) pathway [39], IL-6 bioactivity is associated with higher levels of SARS-CoV-2 detected. In this sense, the presence of viral antigens is related to IL-6-mediated inflammation. However, it has not been reported whether IL-6 inflammation persists in tissues in the later stages of severe COVID-19 when viral titers diminish [40]. In this research, we detected a significant content of IL-1β and IL-6 in the saliva of the V-COVID-19 group at the moment of the PCR diagnosis.

As a perspective, following this research line, we plan to increase the population to discriminate against V-COVID-19 people from the V-Healthy group. Moreover, we are building a portable infrared sensor, employing a quantum cascade diode that analyzes saliva as a biological sample. The sample would be analyzed three times, showing the result on a screen without needing specialized personnel and consumables.

## 5. Conclusions

With these results, we can state that it was possible to detect biochemical changes through FTIR spectroscopy associated to COVID-19 immune response in vaccinated people, once IgG, IgA, IgM, as well as different cytokines, such as IFN-γ, TNF-α, IL-1 β, IL-6, and IL-10, were detected, highlighting that the concentration of the studied molecules expressed a higher content in the V-COVID-19 group than the V-Healthy group, showing statistical significance.

## Figures and Tables

**Table 1:** Information of the population.

Group	Gender		Age (Years)
	Category	Sample	
V-COVID-19	Male	28	52.2 ± 17
	Female	22	
V-Healthy	Male	77	48 ± 15
	Female	58	

**Table 2:** Distribution of the vaccines in the study groups.

Vaccine	V-COVID-19 *n* (%)	V-Healthy *n* (%)
Oxford, AstraZeneca	15 (30)	40 (29.6)
Cansino	12 (24)	39 (28.9)
BioNTech, Pfizer	9 (18)	32 (23.7)
Sputnik V	2 (4)	12 (8.9)
CoronaVac	4 (8)	2 (1.5)
Covaxin	0 (0)	2 (1.5)
Johnson & Johnson	1 (2)	1 (0.7)
Disown	7 (14)	7 (5.2)

**Table 3:** Days elapsed between the last vaccine dose and sampling.

Population	Min–Max (Days)	Median (IQR)
V-COVID-19	17–324	118 (74, 203)
V-Healthy	20–357	120 (77, 185)
Total (N = 130)	17–357	120 (79, 197)

**Table 4:** Symptoms in the studied population.

Symptoms	V-COVID-19 *n* (%)	V-Healthy *n* (%)	*p*
Dyspnea	23 (46)	0 (0)	0.0001
Cough	16 (32)	2 (1.5)	0.0001
Asymptomatic	14 (28)	119 (88.1)	0.0001
Headache	11 (22)	10 (7.4)	0.005
Runny nose	11 (22)	7 (5.2)	0.001
Odynophagia	9 (18)	4 (3)	0.0001
Fever	9 (18)	3 (2.2)	0.0001
Tiredness	9 (18)	2 (1.5)	0.0001
Myalgia	4 (8)	4 (3)	0.135
Nausea	3 (6)	0 (0)	0.004
Arthralgia	1 (2)	4 (3)	0.720
Diarrhea	1 (2)	3 (2.2)	0.926
Anosmia	1 (2)	1 (0.7)	0.462
Ageusia	1 (2)	0 (0)	0.099

**Table 5:** Comorbidities in the studied population.

Comorbidity	V-COVID-19 *n* (%)	V-Healthy *n* (%)	*p*
Hypertension	21 (42)	32 (23.7)	0.015
Diabetes	16 (32)	16 (11.9)	0.001
Obesity	4 (8)	3 (2.2)	0.067
Overweight	3 (6)	3 (2.2)	0.198
Asthma	3 (6)	0 (0)	0.004
COPD	3 (6)	0 (0)	0.004
CKD	3 (6)	2 (1.5)	0.092
No comorbidities	21 (42)	89 (65.9)	0.003
Others	0 (0)	7 (5.18)	0.101

**Table 6:** Laboratory blood test.

Laboratory Blood Tests	*n*	Median (IQR)
**Hematic biometry**		
Leukocytes (103/μL)	45	7.6 (5.9, 10.5)
Neutrophils (103/μL)		5.5 (2.9, 9)
Lymphocytes (103/μL)		1.2 (0.5, 1.7)
Hemoglobin (g/dL)		13.1 (10.2, 14.8)
Platelets (103/μL)		249 (162, 368.5)
**Blood chemistry test**		
Creatinine (mg/dL)	42	1 (0.8, 1.3)
Urea (mg/dL)		36.4 (30, 62.5)
Glucose (mg/dL)		103 (81, 129)
**Serum electrolytes**		
Na (mmol/L)	44	139.5 (137, 142)
K (mmol/L)		4.1 (3.6, 4.6)
**Hepatic-function test**		
ALT (U/L)	29	28 (16.5, 51.5)
AST (U/L)	30	31.5 (21.8, 53)
ALP (U/L)	15	114 (70, 128)
Bilirubin, total (mg/dL)	10	0.8 (0.7, 0.9)
Albumin (g/dL)	13	3.2 (0.3, 3.6)
**Blood gas test**		
pH	29	7.45 (7.4, 7.48)
PaO2 (mmHg)		59 (50, 71.5)
PaCO2 (mmHg)		34 (30.5, 38)
HCO3− (mmol/L)		24.8 (19.3, 28)
Lactate (mmol/L)		1.2 (0.9, 2.3)
**Other**		
CRP (mg/dL)	33	34.2 (7.5, 69.9)
LDH (UI/L)	32	240 (182.3, 391.3)
Fibrinogen (mg/dL)	31	531 (427, 664)
D-dimer (ng/mL)	27	991 (436, 1927)
Ferritin (ng/mL)	17	287 (144, 737.5)

## Data Availability

This article includes all the generated data and the analysis developed in this study.
